# mRNA expression analysis of the SUMO pathway genes in the adult mouse retina

**DOI:** 10.1242/bio.201410645

**Published:** 2015-01-23

**Authors:** Víctor Abad-Morales, Elena B. Domènech, Alejandro Garanto, Gemma Marfany

**Affiliations:** 1 Departament de Genètica, Facultat de Biologia, Universitat de Barcelona, Barcelona, Spain; 2 Centro de Investigación Biomédica en Red de Enfermedades Raras (CIBERER), Instituto de Salud Carlos III, Barcelona, Spain; 3 Institut de Biomedicina de la Universitat de Barcelona (IBUB), Barcelona, Spain; * Present address: Department of Human Genetics, Radboud University Medical Centre, Nijmegen, The Netherlands; and Radboud Institute for Molecular Life Sciences, Radboud University Medical Centre, Nijmegen, The Netherlands.

**Keywords:** SUMO, sumoylation, *In situ* hybridization, mRNA expression levels, retina, light cycle

## Abstract

Sumoylation is a reversible post-translational modification that regulates different cellular processes by conjugation/deconjugation of SUMO moieties to target proteins. Most work on the functional relevance of SUMO has focused on cell cycle, DNA repair and cancer in cultured cells, but data on the inter-dependence of separate components of the SUMO pathway in highly specialized tissues, such as the retina, is still scanty. Nonetheless, several retinal transcription factors (TFs) relevant for cone and rod fate, as well as some circadian rhythm regulators, are regulated by sumoylation. Here we present a comprehensive survey of SUMO pathway gene expression in the murine retina by quantitative RT-PCR and *in situ* hybridization (ISH). The mRNA expression levels were quantified in retinas obtained under four different light/dark conditions, revealing distinct levels of gene expression. In addition, a SUMO pathway retinal gene atlas based on the mRNA expression pattern was drawn. Although most genes are ubiquitously expressed, some patterns could be defined in a first step to determine its biological significance and interdependence. The wide expression of the SUMO pathway genes, the transcriptional response under several light/dark conditions, and the diversity of expression patterns in different cell layers clearly support sumoylation as a relevant post-translational modification in the retina. This expression atlas intends to be a reference framework for retinal researchers and to depict a more comprehensive view of the SUMO-regulated processes in the retina.

## INTRODUCTION

Sumoylation, the covalent conjugation of a small ubiquitin-like modifier (SUMO) to a target protein, is a cell signalling mechanism involved in the regulation of essential cellular and developmental processes such as nucleus-cytoplasm shuttling, apoptosis or transcription (Hayashi et al., 2002; Pichler and Melchior, 2002; Seufert et al., 1995; Wilson and Rangasamy, 2001). This posttranslational reversible process conjugates SUMO by forming an isopeptide bond between the SUMO C-terminus glycine residue and a lysine residue within a consensus motif of the target substrate (Sampson et al., 2001), although non-consensus motif attachments have also been reported (Wilkinson and Henley, 2010).

In mammals there are four SUMO paralogues (SUMO1 to SUMO4). SUMO2 and SUMO3 are almost identical (they differ from each other by only three N-terminal residues), and are able to form chains on substrate proteins through internal lysine residues (Tatham et al., 2001). Contrarily, SUMO1 is attached as a monomer, or acts as a chain terminator on SUMO2/3 polymers (Matic et al., 2008; Okura et al., 1996; Tatham et al., 2001). Finally, SUMO4 isoform has been predicted from genomic data but has not been identified *in vivo* yet (Wei et al., 2008).

Briefly, sumoylation starts with an inactive SUMO precursor that is cleaved at the C-terminus by a SENP (sentrin/SUMO-specific protease) enzyme. The E1 ligase, consisting of a heterodimer of SAE1 (SUMO-activating enzyme E1) and SAE2, activates the SUMO cleaved peptide in an ATP-dependent manner, and transfers it to the active site of UBC9 (ubiquitin-conjugating 9), the unique E2 ligase. This modified UBC9 can directly conjugate SUMO to the consensus sumoylation motif on a target protein, although usually it interacts with an E3 ligase, which will then recognize the final substrate. The E3 ligases act as scaffolds bringing together the SUMO-loaded UBC9 with the target proteins and allowing the conjugation (Flotho and Melchior, 2013). So far, up to 15 different E3 ligases have been identified in mammal genomes (Wilkinson and Henley, 2010). SUMO deconjugation is performed by a family of cysteine proteases, generically named as SENPs. Six SENP members were first described (Wilkinson and Henley, 2010) and very recently three new members, DESI1, DESI2 and USPL1, have been added to the group of SUMO deconjugating enzymes (Schulz et al., 2012; Shin et al., 2012).

The attachment of SUMO moieties to their substrate targets regulates many relevant physiological processes by modulating enzyme activity, activating transcription factors (TFs), shifting protein subcellular localizations, and eventually, determining their substrate fate. Therefore, a detailed expression map of the genes involved in the metabolism of SUMO is fundamental to understand the cellular role of this small peptide in any tissue. Several groups have previously studied the expression levels of some SUMO metabolism enzymes, particularly in neural tissues, and several E3 ligases related to the regulation of neurotransmitter receptors have been shown to be expressed in the retina (Dütting et al., 2011). Global transcriptome analysis by microarray and next generation sequencing have been also reported for the developing mouse retina, mainly focused on the expression differences in comparison with mutant defective strains (Blackshaw et al., 2004; Brooks et al., 2011). However, no exhaustive and comprehensive analysis has been reported for the SUMO pathway genes in the retina to date. On the other hand, several regulatory circadian rhythm genes, as well as transcription factors (TFs) relevant for cone and rod differentiation, such as *NR2E3* and *NRL*, are regulated by sumoylation (Onishi et al., 2009; Roger et al., 2010; Wright et al., 2004), indicating that the SUMO pathway is important in retinal physiological function. Other examples of the SUMO regulatory roles in vision are illustrated by the fact that mutations in the dual Ubiquitin/SUMO E3 ligase *TOPORS* gene cause retinitis pigmentosa in human (Chakarova et al., 2007) (RetNet, http://www.sph.uth.tmc.edu/RetNet/); and the SUMO ligases HDAC4 and TLS have been also involved in photoreceptor survival or apoptosis (Chen and Cepko, 2009; Chen et al., 2011). These cases are probably the tip of the iceberg concerning the function of SUMO in retina.

In this context, we aimed to analyze the mRNA levels and spatial expression pattern of the complete list of SUMO and SUMO pathway enzymes in the mouse retina, as a means to provide an expression atlas, a reference framework for researchers working in retinal diseases and/or SUMO enzymes. This expression map will give useful clues on the function of SUMO during retinal development and physiology, retinal response to light and oxidative stress and the regulation of the retinal circadian clock genes as well as pinpoint new candidate genes for visual disorders.

## MATERIALS AND METHODS

### Ethics statement

All procedures in mice were performed according to the ARVO statement for the use of animals in ophthalmic and vision research, as well as the regulations of the Animal Care facilities (Estabulari de la Facultat de Farmàcia) at the Universitat de Barcelona (UB). All procedures were evaluated and approved by the Animal Research Ethics Committee (Comité Ètic d'Experimentació Animal, CEEA) of the UB (Permit numbers from the Generalitat de Catalunya DAAM 6562 and 7185).

### Animal handling, tissue dissection and preparation of samples

**Fig. 1.**
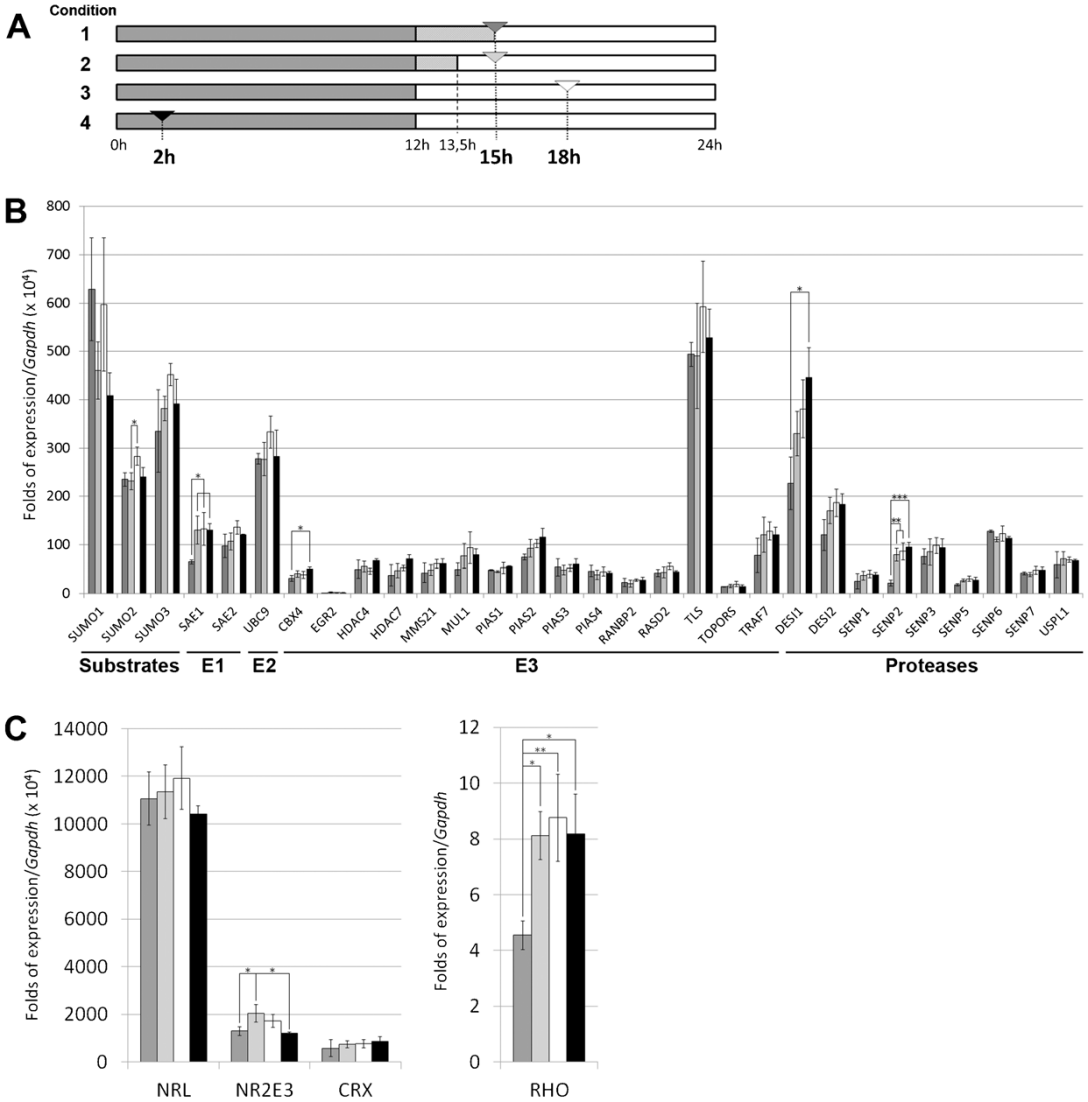
Quantitative relative expression of genes encoding SUMO substrates, SUMO metabolism and other retinal enzymes in the mouse retina. (A) Scheme showing the four conditions of light/dark cycle (grey versus light blocks, respectively) plus the timing of the retinas dissection, which is indicated by an arrowhead: condition 1 (dark grey) – last dark phase lengthened by 3 h (retinas obtained in the dark); condition 2 (light grey) – last dark phase lengthened by 1.5 h plus 1.5 h of exposition to light (retinas obtained under light); condition 3 (white) – retinas obtained in normal light/dark cycle after 6 h of exposition to light; condition 4 (black) – retinas obtained in normal light/dark cycle after 2 h of exposition to dark. (B) Transcriptional levels of SUMO substrate and SUMO metabolism enzyme genes. Levels are obtained as a ratio with *Gapdh* expression (used for normalization) per 10^4^. (C) Transcriptional levels of some relevant retinal genes. *Rhodopsin* levels (right panel) are as high as *Gapdh*, and the ratio is directly represented, whereas the ratio of transcription factors (left panel) is multiplied per 10^4^, as in B. All bars in B and C are coloured indicating the condition under which the retinas were obtained (as explained in A). Three independent retinal cDNA samples (each sample containing 3 different retinas) were analyzed for each of the four conditions. Thus, per each condition 9 retinas from at least 5 different animals and divided in three different samples, were used. Gene expression values are the average of these three samples per condition, and the s.d. bars indicated the variability of expression in the different individuals. The Tukey-Kramer test was used for the multiple comparisons of the condition means. Asterisks (*, ** or ***) show a statistical significant variation (p<0.05, p<0.01 or p<0.001, respectively). The units of expression are directly comparable among genes, except for *Rho*.

Murine retina samples were obtained from P60 C57BL/6J animals. Animals were euthanized by cervical dislocation (with all efforts made to minimize animal suffering) at four different time points and light conditions (as specified in Fig. 1) and retinas were dissected and immediately frozen. Housing under 12:12 h light/dark cycle (LD) is usually considered normal conditions. For conditions 1 and 4, animals were kept in the dark and their retinas were collected in a dark chamber with dim red light, as mice lack the opsins capable to detect this wavelength (L-cones). For *in situ* hybridization, retinas were obtained from animals reared under normal LD cycle and after 6 h of exposition to light (condition 3 of Fig. 1A), collected and treated as described elsewhere (Garanto et al., 2012).

### RNA extraction and cDNA generation

For each of the four conditions, three independent samples were analyzed, and each sample contained three frozen retinas (of two different animals). Thus, per condition, nine retinas were analyzed in a total of three different samples. The retinas were homogenized using a Polytron PT1200E homogenizer (Kinematica, AG, Lucerne, Switzerland). For total RNA extraction, the High Pure RNA Tissue Kit (Roche Diagnostics, Indianapolis, IN) was used, following the manufacturer's instructions with minor modifications (longer treatment with DNAseI). Reverse transcription reactions were carried out using the qScript™ cDNA Synthesis Kit (Quanta BioSciences, Inc., Gaithersburg, MD).

### qRT-PCR

Quantitative reverse transcription PCR was performed using the LightCycler® 480 SYBR Green I Master and a LightCycler® 480 Multiwell Plate 384 (Roche Applied Science) in a final reaction volume of 10 µl. Raw data were analyzed with the LightCycler® 480 software using the Advanced Relative Quantification method. *Gapdh* expression was used to normalize the levels of expression. *Rho* and *Cerkl* were considered as reference genes with high and low levels of expression in the mouse retina, respectively. Given that RT-PCR is a very sensitive technique, three technical replicates of the same sample were performed for each gene to minimize procedure errors. The specific gene primers used are listed in supplementary material Table S1. The error bars correspond to the s.d. of the expression levels detected in the three biological samples, depicted in Fig. 1B,C. After testing for equal s.d. and normal distribution in the different conditions (Bartlett and Shapiro Wilk tests, respectively), a multiple comparison analysis of the means in the different conditions was performed using a Tukey-Kramer test to assess statistical significance.

### *In situ* hybridization

For *in situ* hybridization, cDNA fragments (400–500 bp) of each gene were subcloned into pGEM-T® Easy Vector (Promega), and sense/antisense riboprobes were generated from the flanking T7 or SP6 RNApol promoters. Eighteen micrometre sections were recovered on either custom made poly-lysine covered slides, or commercial Superfrost Plus glass slides (Electron Microscopy Sciences, Hatfield, PA), dried 1 h at RT, rinsed three times for 10 min with phosphate-buffered saline (PBS), treated with 2 µg/ml proteinase K for 15 min at 37°C, washed twice for 5 min with PBS, and postfixed with 4% PFA. Acetylation with 0.1 M triethanolamine-HCl (pH 8.0) containing first 0.25%, and then 0.5% acetic anhydride, was performed for 5 min each. Hybridization was carried out overnight at 55°C with digoxigenin-labeled riboprobes (2 µg/ml ) in 50% formamide, 1×Denhardt's solution, 10% dextran-sulfate, 0.9 M NaCl, 100 mM Tris-HCl (pH 8.0), 5 mM EDTA (pH 8.0), 10 mM NaH_2_PO_4_, and 1 mg/ml yeast tRNA.

After hybridization, slides were washed in 2× SSC for 20 min at 55°C, equilibrated in NTE (0.5 M NaCl, 10 mM Tris-HCl pH 8.0, 5 mM EDTA) at 37°C, and then treated with 10 µg/ml RNase A in NTE at 37°C for 30 min. Subsequently, sections were washed at 37°C in NTE for 15 min, twice in 2× SSC and 0.2× SSC for 15 min each, equilibrated in Buffer 1 (100 mM Tris-HCl pH 7.5, 150 mM NaCl), and blocked in Blocking Buffer (1% BSA and 0.1% Triton X-100 in buffer 1) for 1 h. An anti-digoxigenin-AP conjugate antibody (1:1000; Roche Diagnostics, Indianapolis, IN) in Blocking Buffer was incubated overnight at 4°C. Sections were then washed twice in Buffer 1 for 15 min, Buffer 2 (100 mM Tris-HCl pH 9.5, 150 mM NaCl), and Buffer 2 supplemented with 50 mM MgCl_2_ for 5 min each, prior to exposure to BM Purple AP Substrate (Roche Diagnostics, Indianapolis, IN). Reactions were stopped by washing in 1× PBS. Sections were cover-slipped with Fluoprep (Biomérieux, France) and photographed using a Leica DFC Camera connected to a Leica DM IL optic microscope (Leica Microsystems, Germany).

### Retinal explants

Freshly enucleated eyes from mice were manipulated in Leibovitz L15 media, and the retinas were dissected from the pigmented epithelium and other ocular structures. Careful cuts at the edges were performed to flatten the tissue and each retina was separately placed with the photoreceptors upside in a 6 well plate with Neurobasal media (plus 1% penicillin/streptomycin, 2% l-glutamine, 7.46% glucose, 16.66% NaH_2_CO_3_, 2% B27) on top of a 0.4 µm transwell membrane (30 mm Diameter, Millicell®Cell Culture Inserts). All retinas were kept overnight at 37°C and 5% CO_2_ in the dark. One retina from each individual (n = 6) was exposed to light for 1.5 h while the other remained in the dark (conditions 2 and 1, respectively). After the treatment, all retina explants were individually frozen in liquid nitrogen.

### Protein lysates and immunodetection by western blot

Each retinal explant was separately homogenized in RIPA buffer (Desoxycolate 0.25% w/v, NP40 1% v/v, Tris pH 7.5 1 M, EDTA 500 mM, NaCl 5 M and protease inhibitor). Protein lysates were loaded on 10–12.5% SDS-PAGE gels, transferred onto PVDF membranes and blocked with 5% BSA in PBST for 1 h. Primary antibodies (1:500 dilution) against TLS (Biorbyt, Cambridge, UK), CRX (Abnova, Taiwan) and NR2E3 (Abcam, Cambridge, UK) were incubated overnight at 4°C, and then with secondary antibodies (dilution 1:2000) for 1 h. For normalization and quantification, immunodetection (dilution 1:1000) against either GAPDH (Abcam, Cambridge, UK) or α-Tubulin (Sigma-Aldrich, St. Louis, MO) were used. The ImageJ software (http://imagej.nih.gov/ij/) was used for quantification of the bands, followed by statistical analysis according to a Student's t-test applied after testing for equal s.d. and normal distribution (Bartlett and Shapiro-Wilk tests, respectively).

## RESULTS

### Expression levels of SUMO and SUMO pathway enzymes in the mouse retina

Given that mouse is one of the animal models of choice to study the retina, and that the human SUMO pathway genes have orthologues in mouse, we aimed to assess the relative mRNA expression levels of all known SUMO metabolism enzymes and provide a preliminary map of their expression pattern in the adult murine retina. Considering that the SUMO pathway is relevant for the regulation of the circadian rhythm, we also addressed whether exposure to light or to darkness caused a transcriptional effect (induction or repression) in the genes of this pathway. We analyzed three independent retinal samples per condition and each sample contained three retinas from different P60 mice reared in 12:12 h LD. In total, per each condition we used 9 retinas from at least 5 different animals, divided in three different samples. The four conditions considered were (Fig. 1A) namely: condition 1 – after 15 h of dark phase (in normal conditions the animals would already have been exposed to daylight for 3 h); condition 2 – after 13.5 h of dark phase followed by 1.5 h of light (to detect quick transcriptional activation/inhibition peaks due to light exposition and compare directly with the previous case); condition 3 – in normal daylight phase (after 6 h of daylight), and finally, condition 4 – in normal dark phase (after 2 h of darkness, to detect transcriptional activation/inhibition due to darkness exposure or darkness circadian phase). In order to distinguish differences in gene expression due to direct light exposition from those generated by circadian rhythmicity, we compared retinas obtained at the same time of the day, after 1.5 h of light exposition (condition 2) compared to the retinas obtained in sibling mice kept during all this time in the dark after the night cycle (condition 1). In these circumstances, any gene whose expression appears modified in the light versus the dark (condition 2 compared to 1) might reflect the effect of light exposition irrespective of the circadian rhythm (which will be the same for the retinas in these two conditions). Conditions 3 and 4 were considered as normal light/dark cycle time-points to illustrate transcriptional values under circadian rhythmicity.

Quantitative RT-PCRs were performed to assess the expression levels for the analysis of 30 genes (the designed primer pairs are listed in supplementary material Table 1), including the reported SUMO E1, E2, E3 ligases and proteases, plus the SUMO substrates (Fig. 1B). Moreover, we also analyzed a highly expressed retinal gene that has been reported to show circadian rhythmicity (*Rho*) (von Schantz et al., 1999), plus three transcription factors that are relevant for cone-rod fate and function and might be sensitive to light/dark conditions (Fig. 1C). Two relevant genes involved in retinal dystrophies, the previously mentioned *Rho* and *Cerkl*, were also included as internal reference threshold values for high and low levels of gene expression in the retina, respectively.

All the values were normalized with respect to the expression of *Gapdh* and multiplied per 10^4^ (except for *Rho*, which is expressed four to five orders of magnitude higher), and thus, the relative values of expression can be directly compared. Fig. 1 shows the mean value and the s.d. of three different biological samples per gene and condition, where each sample contained at least 3 retinas. Per gene and sample, every value was obtained by triplicate (average of three technical replicates) to increase data robustness.

The three genes encoding the SUMO1, SUMO2 and SUMO3 substrates were highly expressed. Notably, the gene for the only E2 enzyme (*Ubc9*) was more highly expressed than the genes coding for the two components of the E1 ligase heterodimer (*Sae1* and *Sae2*). Taken together, the group of E3 ligases and SUMO proteases showed a higher variability in the levels of expression. The most highly expressed E3 ligase gene was *Tls*, in contrast to the basal expression of *Egr2* (around two orders of magnitude lower). Among the deconjugating enzymes, *Desi1* was the most highly expressed, whereas *Senp1* and *Senp5* showed low levels of expression. Concerning the analysis of relevant retinal genes, *Rho* was the most highly expressed, directly comparable to *Gapdh*, followed by *Nrl* and finally *Nr2e3* and *Crx*.

**Fig. 2.**
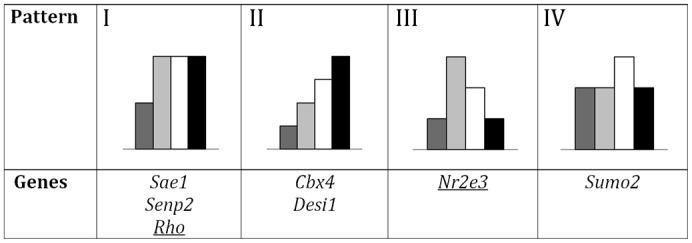
Classification of the SUMO-pathway and other retinal genes according to their pattern of expression activation/inhibition in mouse under several light-dark conditions. Some SUMO metabolism and other relevant retinal genes were grouped with statistical significance by the similarity in their patterns of expression, according to the qRT-PCR. Note that the first three patterns show an activation by light exposition (condition 1 versus 2) that is further increased (I), maintained (II) or decreased (III) in the following conditions (3 and 4), while pattern IV showed an isolated increase in the third condition. Histogram colouring is as in Fig. 1.

Although most of the gene transcription levels remained unaltered in the different light/dark conditions, a few of them were consistently and significantly altered. As shown in Fig. 2, *Sae1*, *Senp2* and *Rho* (pattern I) showed a quick activation peak when exposed to light (condition 1 versus 2) sustained during the light and early dark phase (conditions 3 and 4). Similarly, *Cbx4* and *Desi1* (pattern II) had a constant increase of expression from condition 1 to 4. The retinal transcription factor *Nr2e3* (pattern III) showed a sharp peak in response to light exposition (condition 2) that steadily decreased to the initial levels (condition 4 and 1). Finally, the expression of *Sumo2* (pattern IV) increased during daylight (third condition) and was slightly lower in the rest of conditions. Although some other behaviour patterns could be observed, the expression values were too variable to be statistically significant (e.g. *Crx* showed a lower transcript level in condition 1 for some samples compared to the rest of conditions, suggesting transcriptional activation when exposed to light).

### Spatial expression pattern of the SUMO pathway genes

**Fig. 3.**
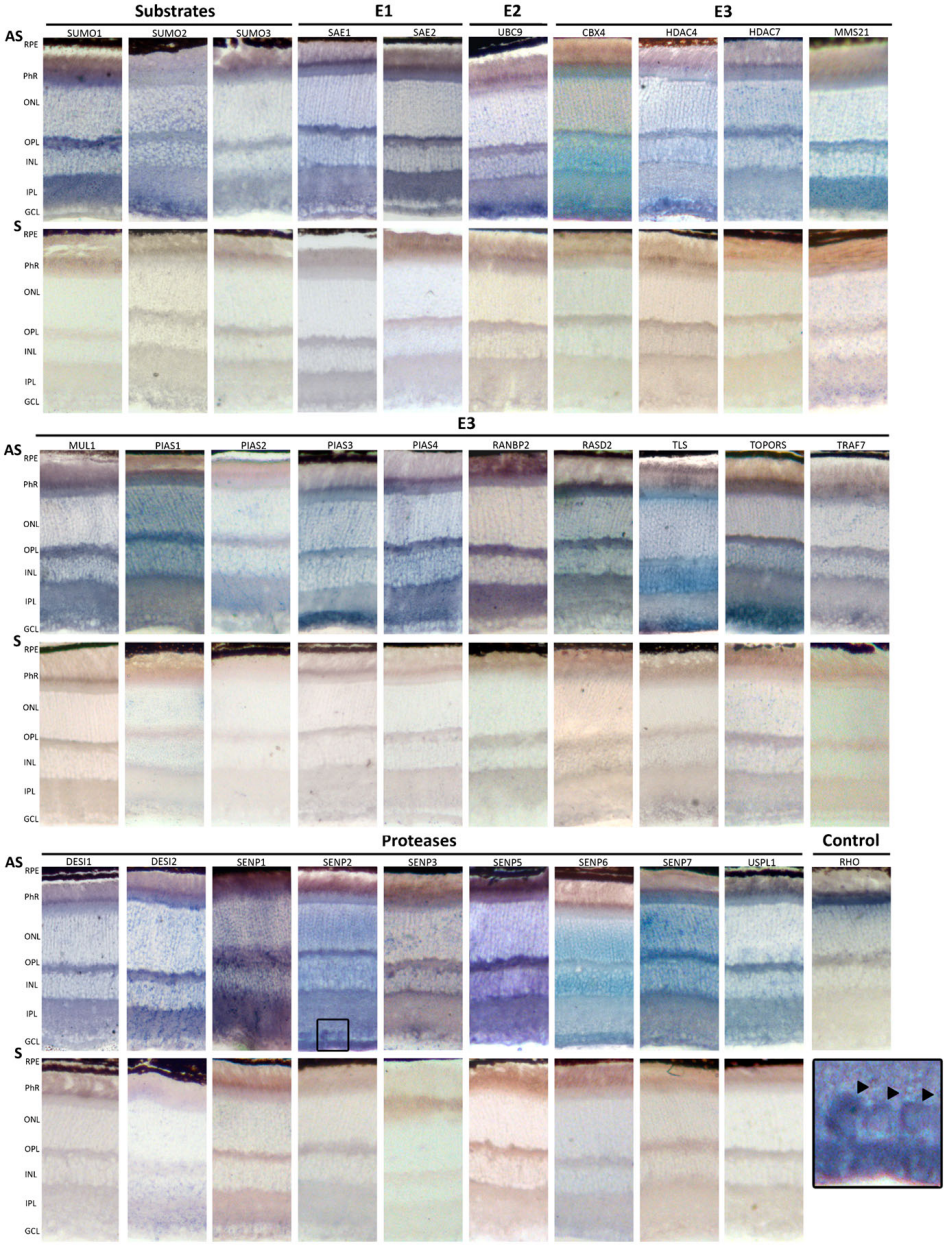
*In situ* hybridization on murine retina cryosections of the genes encoding SUMO substrates and SUMO E1, E2, and E3 ligases, and proteases. Representative images obtained after the *in situ* hybridization of Antisense (AS) and Sense (S) digoxigenin-labelled riboprobes, stained for the same period of time per each gene. Antisense riboprobes reflect the pattern of gene expression, whereas the sense probes are the corresponding negative controls. The antisense *Rhodopsin* probe, which strongly labels the inner photoreceptor segment, was used as a positive control for the assay. RPE, Retinal Pigment epithelium; PhR, Photoreceptor cell layer; ONL, outer nuclear layer; OPL, Outer plexiform layer; INL, Inner nuclear layer, IPL, inner plexiform layer; GCL, ganglion cell layer. The boxed region at the bottom right is an amplification of the *Senp2 in situ* hybridization at the GCL level. The black arrowheads indicate nuclear/perinuclear mRNA localization.

**Fig. 4.**
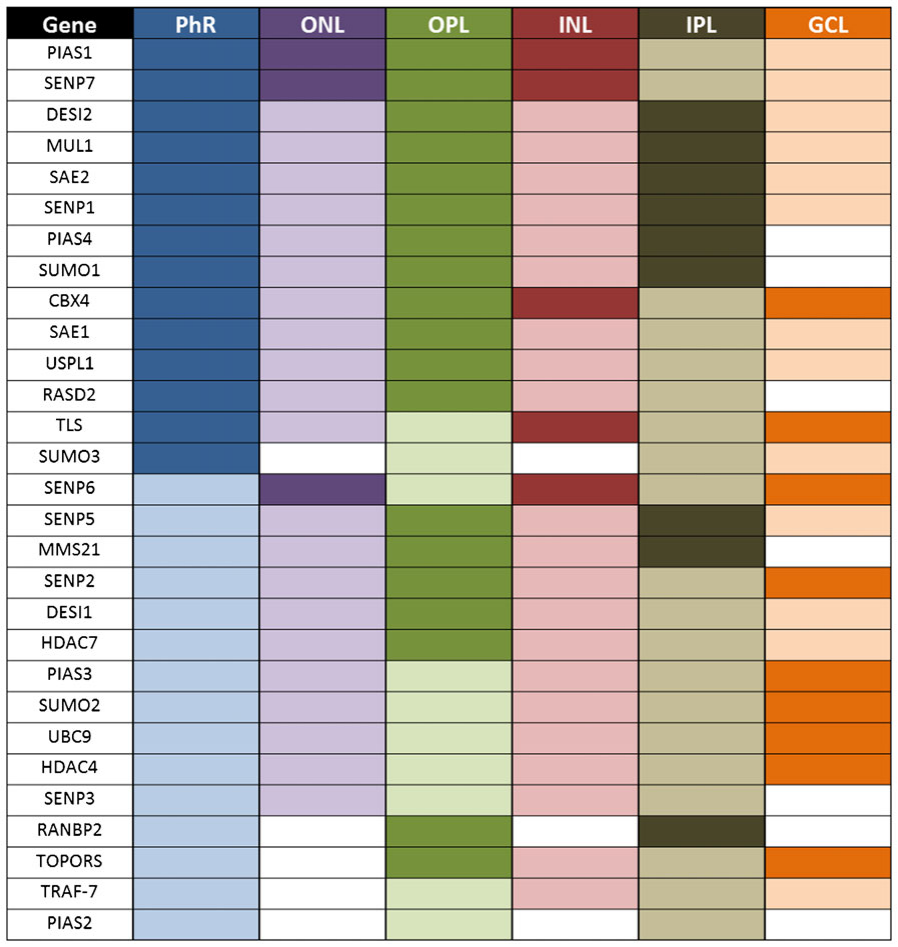
Summarized graphic representation of the mRNA pattern of the SUMO pathway genes as revealed by *in situ* hybridization on murine retina cryosections. Colour intensity in this figure reflects *in situ* hybridization signal intensity per each gene by direct comparison of the signal rendered in different layers in the same retinal preparation. Intensities are not directly comparable among different genes, as each *in situ* hybridization required different incubation times. For each gene, sense (negative control) and antisense riboprobes were always processed in parallel and following the same incubation times. This graphic interpretation depicts the mRNA positive signal per gene and layer after deducting the corresponding negative signal (if any). Phr, Photoreceptor cell layer; ONL, outer nuclear layer; OPL, Outer plexiform layer; INL, Inner nuclear layer, IPL, inner plexiform layer; GCL, ganglion cell layer.

In order to localize the expression of SUMO substrates and enzymes in the mouse retina, we resorted to *in situ* hybridization as a means to obtain a comprehensive atlas of expression irrespective of the availability of specific and sensitive commercial antibodies for all the set of proteins. Antisense riboprobes (with their corresponding sense riboprobes) were generated for each gene and hybridized on adult (P60) retinal sections obtained during light time (condition 3 of Fig. 1A). *Rhodopsin*, a highly expressed gene in the inner segment (IN) of rod photoreceptors, was used as a positive control. For each gene, the ISH of antisense and sense (negative control) riboprobes were processed in parallel so that the signal obtained was considered specific for the mRNA localization of every assayed gene, and the relative intensity of the mRNA detection within the same retina for each gene is comparable. Representative *in situ* hybridization images for all the genes are shown in Fig. 3. In addition, a qualitative schematic summary of the mRNA localization data per layer and gene, grouped by expression pattern, is shown in Fig. 4.

The expression of *Sumo1*, *Sumo2* and *Sumo3* was rather ubiquitous, although some differences could be observed. *Sumo1* was highly expressed in the photoreceptors inner segment and in the plexiform layers, and not detected in the GCL, where *Sumo2* showed a high signal; while *Sumo3* expression was not detected in the nuclear layers. Consistent with their instrumental function in the SUMO pathway, the E1 ligase genes, *Sae1* and *Sae2*, and the unique E2 *Ubc9* shared a similar ubiquitous pattern, although *Ubc9* showed a stronger signal in the GCL.

Not unexpectedly, the proteases and E3 ligases showed a wide variety of expression patterns (Figs 3 and 4). The inner segment of photoreceptors showed expression signal for all genes. The signal is particularly high for *Cbx4*, *Desi2*, *Mul1*, *Pias1*, *Pias4*, *Rasd2*, *Senp1 Senp7*, *Tls* and *Uspl1*. Notably, both outer and inner nuclear layers showed a very similar pattern of hybridization, and perinuclear signal was observed for most genes. *Pias1*, *Senp6* and *Senp7* produced a stronger signal in both ONL and INL, whereas *Tls* only did in the INL. On the contrary, *Pias2* and *RanBP2* produce nearly undetectable signal in both nuclear layers. Remarkably, high mRNA localization signal for most genes was observed in the two plexiform layers, particularly in the outer plexiform layer, in clear contrast with the corresponding sense riboprobes (negative controls). Of note, the GCL rendered a very distinctive pattern of mRNA hybridization, in clear contrast to that observed in the photoreceptor inner segment. The GCL showed high expression of *Cbx4*, *Hdac4*, *Pias3*, *Senp2*, *Senp6*, *Tls* and *Topors*, whereas no – or weak – signal was observed for *Mms21*, *Pias2*, *Pias4*, *RanBP2*, *Rasd2* and *Senp3*.

Considering the pattern produced per genes (Fig. 4), *Pias2* and *RanBP2* mRNA localization was extremely weak in the retina layers with a significant number of nuclei (ONL, INL, GCL). *Tls* mRNA followed a unique pattern and was strongly expressed and localized in the photoreceptors (inner segment), the INL and GCL (Figs 3 and 4). Finally, it might be relevant to note that *Senp2* mRNA was localized nuclearly/perinuclearly in some cells at the GCL, a localization that was not observed for any other SUMO-pathway gene mRNA (see black arrows in amplified section in Fig. 3). We selected several genes to perform *in situ* hybridizations in mouse retinas obtained in conditions 1 (dark) and 2 (exposure to light) to compare them with those obtained in condition 3 (light). No detectable changes in the mRNA localization in the retinal cell layers were apparent for the tested genes (data not shown).

### Differential gene expression induced by light exposition

**Fig. 5.**
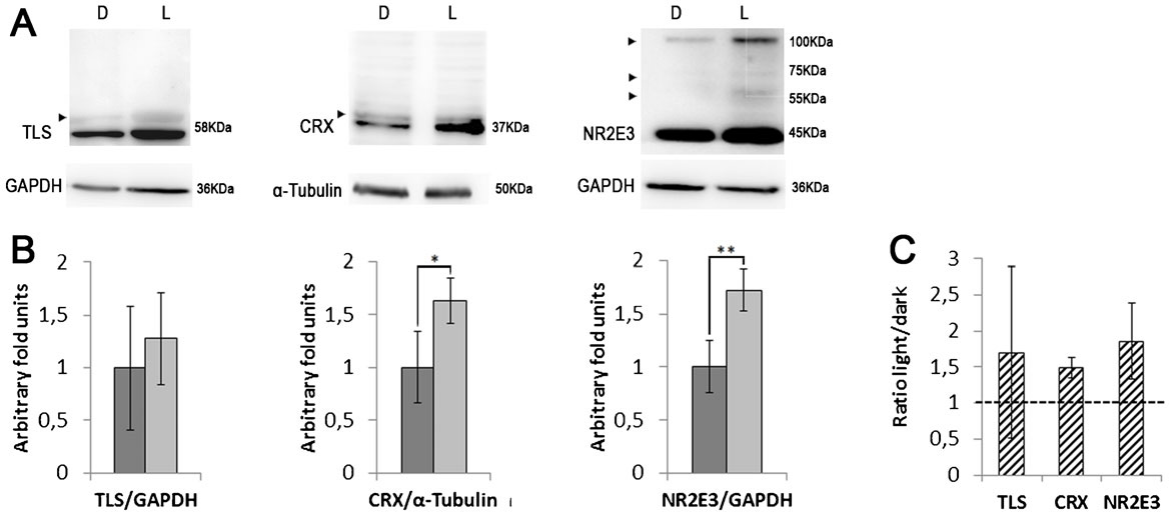
Immunodetection of several proteins in retinal explants under dark or light conditions. (A) Western blots of TLS, CRX, and NR2E3 in retinal explants of single individuals in which one retina was maintained in the dark (D, condition 1), while the other counterpart was exposed to light for 90 min after dark (L, condition 2). This is a representative image of n = 6. Immunodetection of GAPDH or α-tubulin was used as a normalization control. Arrowheads indicate higher molecular weight bands compatible with post-translational modifications, such as sumoylation. (B) Protein level quantification of the retinal explants in dark (1) and light (2) conditions (dark and light grey, respectively). Bars indicate s.d. (n = 6). Fold-induction is in arbitrary units, considering the mean value of the protein level in the dark as 1. Statistical significance is indicated by * (p<0.05) and ** (p<0.01) according to a Student's t-test, assuming normality (after Bartlett and Shapiro-Wilk tests). (C) Fold induction of the protein expression levels between the light vs dark conditions in the retinal explants from the same animal. For each pair of explants, the expression level in the dark was arbitrarily considered as the unity to allow direct comparison.

To further investigate the possible effect of light induction in gene expression, retinal explants from several individuals were performed, in which after the night cycle one of the retinas remained in the dark (condition 1) while the other was exposed to light for 1.5 h (condition 2). The lysates from the two retinas per animal were electrophoresed and immunodetected simultaneously for direct comparison. The protein levels of TLS (the most highly expressed gene of the SUMO pathway) as well as the transcription factors CRX and NR2E3, which showed a transcriptional activation after light exposition by qRT-PCR, were analyzed (Fig. 5A). Note that these three proteins produce at least one weak mirror band of higher molecular weight size when immunodetected by Western blot, compatible with post-translational modifications, such as sumoylation (arrowheads in Fig. 5A). In fact, sumoylation of NR2E3 has already been reported (Onishi et al., 2009).

According to our results in retinal explants (n = 6 animals), the protein levels of CRX and NR2E3 were different in the dark and light conditions. Remarkably, CRX levels were 1.5 fold higher in the light and NR2E3, 1.8 fold (statistical significance of p<0.05 and p<0.01 respectively). TLS instead showed a high variability in the protein expression levels, but no statistically significant differences were found comparing the explants in the two conditions (Fig. 5B). The fold values obtained from comparing the protein levels in the explants of light and dark per animal were also consistent with these results (Fig. 5C), pointing to light induction.

## DISCUSSION

Most work on the functional relevance of SUMO has focused on cell cycle, DNA repair and cancer in cultured cells, and data on the inter-dependence of separate components of the SUMO pathway in highly specialized tissues are largely absent. Therefore, reports on the relevance of SUMO in retina are scanty and most of the published findings relate to sumoylation of particular substrates, such as TFs, neurotransmitter receptors or circadian clock regulators. Thus, a systematic descriptive analysis of the SUMO metabolism gene expression in the retina was missing. The present work aims to fill this void, and by reporting mRNA expression profiles and localization in the different adult mouse retinal layers – under four different conditions at several times of the light/dark cycle – provide a useful picture in which to locate the modifier of a protein of interest concerning the complex circuitry of the vision process.

The qRT-PCR detected the expression of all the SUMO substrates and enzymes in the mouse retina. As expected, the genes encoding the substrates, *SUMO1* to *3*, showed high expression levels due to their central role in sumoylation. The genes coding for the two E1 ligases, *Sae1* and *Sae2*, known to act as a heterodimer, were comparably expressed; and *Ubc9*, encoding the unique E2 conjugating enzyme, showed one of the highest levels of expression among the analyzed genes. The wide expression range for SUMO E3 ligases and proteases suggest different retinal roles/relevance, as these enzymes display target specificity and in most cases, non-redundancy. The least and the highest expressed SUMO pathway genes were among the E3 ligases: *Egr2* (also known as the transcription factor *Krox20*) and *Tls*, respectively (approximately 3 orders of magnitude difference in mRNA expression levels, arbitrary units, Fig. 1B). In this context, it is worth noting that *Tls* (one of the genes causing amyotrophic lateral sclerosis in human) had been already the focus of previous works in the retina, given that its overexpression led to progressive photoreceptor degeneration in *Drosophila* (Chen et al., 2011), and that TLS is involved in the regulation of the receptor of the NMDA neurotransmitter in rat ganglion retinal cells (Selamat et al., 2009). Other genes previously reported to play relevant roles in this tissue showed comparable medium-low level of expression: *Hdca4*, a promoter of retinal cells survival (Chen and Cepko, 2009); *Topors*, a causative gene of retinitis pigmentosa in humans (Chakarova et al., 2007), and *Pias3*, encoding the SUMO E3 ligase that regulates the repressor function of NR2E3 in mouse cone genes (Onishi et al., 2009). Finally, the SUMO proteases were more homogeneously expressed, with differences in expression not larger than one order of magnitude. Notably, the more highly expressed proteases corresponded to two newly incorporated genes, *Desi1* and *Desi2* (Shin et al., 2012), indicating that their function might be important in the retina.

Focusing on the three TFs relevant for photoreceptor fate, their levels of expression were higher than the genes of the SUMO pathway, probably due to their central role in regulating genes for photoreceptor function and maintenance. *Crx* and *Nr2e3* showed similar levels of expression, whereas *Nrl* (one of the genes controlling rod fate) was the most highly expressed (around ten folds the expression of *Crx*), consistent with the high number of rods in the murine retina.

Gene expression in the mouse retina had been previously profiled either during embryonic and postnatal retinal development using serial analysis of gene expression (SAGE) and microarrays (Blackshaw et al., 2004), or comparing the retinal transcriptome of wild-type and *Nrl*^−/−^ mice by next generation sequencing (NGS) (Brooks et al., 2011). These reports focused on the identification of upregulated or downregulated genes during these processes or genetic conditions and, except for *Pias1* and *Pias3* during retinal development, no SUMO pathway gene showed any major difference in its expression. These apparent stasis may be explained by maintenance of the transcription levels during retinal development for most of the SUMO pathway enzymes, or probably, that subtle differences (less than two fold, for instance) might be missed in high-throughput analysis due to very stringent cut-off values, and might stand out in smaller scale and more targeted transcriptional studies, such as the work presented here. Indeed, the regulation of SUMO genes function might also rely on mechanisms other than transcription, such as protein stability or activity regulation by post-translational modifications (e.g. many SUMO pathway enzymes show retroactive and crosstalk regulation by SUMO and ubiquitin (Denuc and Marfany, 2010)).

When focusing on the expression in the four different light/dark conditions, SUMO machinery seemed to remain mainly unaltered. Only *Sumo2*, *Sae1*, *Cbx4*, *Desi1* and *Senp2* showed statistical significant differences in the expression levels in some of the four conditions. SAE1 is known to act as a heterodimer together with SAE2, and the induction of *Sae1* observed after light exposition might reflect a different regulation for these two genes. In the entire set of ligase genes only *Cbx4* showed significant variations, with maximum level of transcription at night (condition 4), while in the protease group, formed by fewer genes, *Desi1* and *Senp2* were the only ones to show differential expression levels in light versus dark conditions. In summary, four different patterns of expression emerged. One possible explanation for these results would be that sumoylation as a posttranslational modification could be regulating (or regulated by) the response to light exposition.

As the analyzed TFs are direct transcriptional regulators of downstream target genes, the differences in expression due to light exposition might be relevant. A small increase or decrease in their mRNA and protein levels, even their interaction in activating or inhibiting complexes might cause a significant cascade effect for the physiology of the retina. In this context, it is worth noting that the increase in mRNA levels after light exposition has been confirmed for *Crx* and *Nr2e3* by their protein relative levels in retinal explants. The differences in transcription were amplified in both cases at the protein level (for instance *Nr2e3* transcript levels were increased 1.4 fold when comparing condition 2 (light) to 1 (dark), while the protein levels were 1.8 fold higher). On the other hand, no apparent difference in the sumoylation state of the protein NR2E3 could be observed between these two conditions. Further work may reveal the relevance of this increase of expression when the retinas are exposed to light. Finally, *Rho* (whose expression is regulated by the above transcription factors) follows circadian rhythmicity and has been reported to show a sharp transcriptional peak at the starting time of the dark phase (von Schantz et al., 1999). Conditions 3 and 4 in this work overlapped this exact timing and did not exactly coincide with this sharp transcriptional induction. We cannot discard that a similar sharp transcriptional response during daily light/dark cycling went undetected by our analysis in any of the studied genes.

Concerning mRNA localization, *in situ* hybridization is not a direct quantitative assay, nonetheless several signal intensities (high, low and non-detectable signal) were visible for each gene in the retinal layers (Fig. 4). Most genes showed a ubiquitous signal, including plexiform and nuclear layers, pointing to a basal function in most cells. Notably, all the analyzed genes were expressed in photoreceptors, and the mRNA mainly localized in the inner segment, where most of the translational machinery resides. More remarkable was the detection of high levels of mRNA expression in the plexiform layers, pointing to localization in the synaptic processes and buds (the low resolution of *in situ* hybridization did not allow a more accurate assignment). Although these results should be with some caution until further work refine this localization and its physiological relevance, there are increasing evidences of specific mRNA localization along the axons and in the synapses, forming ribonucleoprotein complexes (RNPs, P-bodies, stress granules, etc.) (Liu-Yesucevitz et al., 2011). The E3 ligase *Pias3* is one of the few SUMO pathway genes previously studied in the retina, and previous reports detected high *Pias3* mRNA expression in developing photoreceptors whereas the signal seemed to decrease in the adult retina (Blackshaw et al., 2004). On the other hand, other reports detected the PIAS3 protein in the GCL and in the photoreceptor outer segment in the mature retina (Dütting et al., 2011), in accordance to its role as a NR2E3 regulator. In this work, we also detected *Pias3* mRNA in the adult retina (P60) throughout all retinal layers but more strongly at the GCL (Figs 3 and 4).

In summary, the wide expression of the SUMO pathway genes and the diversity of detected expression patterns in the different retinal cell layers and light/dark conditions in mouse – added to other previous reports on particular genes and targets – clearly supports sumoylation as a relevant post-translational modification in this tissue. Since one of the emerging concepts is the requirement for simultaneous SUMO conjugation/deconjugation of multiple protein targets involved in the same biological process, within dynamic post-translational complexes (Flotho and Melchior, 2013; Psakhye and Jentsch, 2012), this mRNA expression atlas intends to be a reference framework for retinal researchers and may contribute to depict a more comprehensive view of the SUMO-regulated processes in the retina.

## Supplementary Material

Supplementary Material

## References

[b1] Blackshaw S. Harpavat S. Trimarchi J. Cai L. Huang H. Kuo W. P. Weber G. Lee K. Fraioli R. E. Cho S. H. (2004). Genomic analysis of mouse retinal development. PLoS Biol. 2, e247 10.1371/journal.pbio.0020247 15226823 PMC439783

[b2] Brooks M. J. Rajasimha H. K. Roger J. E. Swaroop A. (2011). Next-generation sequencing facilitates quantitative analysis of wild-type and Nrl(−/−) retinal transcriptomes. Mol. Vis. 17, 3034–3054. 22162623 PMC3233386

[b3] Chakarova C. F. Papaioannou M. G. Khanna H. Lopez I. Waseem N. Shah A. Theis T. Friedman J. Maubaret C. Bujakowska K. (2007). Mutations in TOPORS cause autosomal dominant retinitis pigmentosa with perivascular retinal pigment epithelium atrophy. Am. J. Hum. Genet. 81, 1098–1103. 10.1086/521953 17924349 PMC2265653

[b4] Chen B. Cepko C. L. (2009). HDAC4 regulates neuronal survival in normal and diseased retinas. Science 323, 256–259. 10.1126/science.1166226 19131628 PMC3339762

[b5] Chen Y. Yang M. Deng J. Chen X. Ye Y. Zhu L. Liu J. Ye H. Shen Y. Li Y. (2011). Expression of human FUS protein in Drosophila leads to progressive neurodegeneration. Protein Cell 2, 477–486. 10.1007/s13238-011-1065-7 21748598 PMC3563268

[b6] Denuc A. Marfany G. (2010). SUMO and ubiquitin paths converge. Biochem. Soc. Trans. 38, 34–39. 10.1042/BST0380034 20074031

[b7] Dütting E. Schröder-Kress N. Sticht H. Enz R. (2011). SUMO E3 ligases are expressed in the retina and regulate SUMOylation of the metabotropic glutamate receptor 8b. Biochem. J. 435, 365–371. 10.1042/BJ20101854 21288202

[b8] Flotho A. Melchior F. (2013). Sumoylation: a regulatory protein modification in health and disease. Annu. Rev. Biochem. 82, 357–385. 10.1146/annurev-biochem-061909-093311 23746258

[b9] Garanto A. Vicente-Tejedor J. Riera M. De la Villa P. Gonzàlez-Duarte R. Blanco R. Marfany G. (2012). Targeted knockdown of Cerkl, a retinal dystrophy gene, causes mild affectation of the retinal ganglion cell layer. Biochim. Biophys. Acta 1822, 1258–1269. 10.1016/j.bbadis.2012.04.004 22549043

[b10] Hayashi T. Seki M. Maeda D. Wang W. Kawabe Y. Seki T. Saitoh H. Fukagawa T. Yagi H. Enomoto T. (2002). Ubc9 is essential for viability of higher eukaryotic cells. Exp. Cell Res. 280, 212–221. 10.1006/excr.2002.5634 12413887

[b11] Liu-Yesucevitz L. Bassell G. J. Gitler A. D. Hart A. C. Klann E. Richter J. D. Warren S. T. Wolozin B. (2011). Local RNA translation at the synapse and in disease. J. Neurosci. 31, 16086–16093. 10.1523/JNEUROSCI.4105-11.2011 22072660 PMC3241995

[b12] Matic I. van Hagen M. Schimmel J. Macek B. Ogg S. C. Tatham M. H. Hay R. T. Lamond A. I. Mann M. Vertegaal A. C. (2008). In vivo identification of human small ubiquitin-like modifier polymerization sites by high accuracy mass spectrometry and an in vitro to in vivo strategy. Mol. Cell. Proteomics 7, 132–144. 10.1074/mcp.M700173-MCP200 17938407 PMC3840926

[b13] Okura T. Gong L. Kamitani T. Wada T. Okura I. Wei C. F. Chang H. M. Yeh E. T. (1996). Protection against Fas/APO-1- and tumor necrosis factor-mediated cell death by a novel protein, sentrin. J. Immunol. 157, 4277–4281. 8906799

[b14] Onishi A. Peng G. H. Hsu C. Alexis U. Chen S. Blackshaw S. (2009). Pias3-dependent SUMOylation directs rod photoreceptor development. Neuron 61, 234–246. 10.1016/j.neuron.2008.12.006 19186166 PMC2701228

[b15] Pichler A. Melchior F. (2002). Ubiquitin-related modifier SUMO1 and nucleocytoplasmic transport. Traffic 3, 381–387. 10.1034/j.1600-0854.2002.30601.x 12010456

[b16] Psakhye I. Jentsch S. (2012). Protein group modification and synergy in the SUMO pathway as exemplified in DNA repair. Cell 151, 807–820. 10.1016/j.cell.2012.10.021 23122649

[b17] Roger J. E. Nellissery J. Kim D. S. Swaroop A. (2010). Sumoylation of bZIP transcription factor NRL modulates target gene expression during photoreceptor differentiation. J. Biol. Chem. 285, 25637–25644. 10.1074/jbc.M110.142810 20551322 PMC2919127

[b18] Sampson D. A. Wang M. Matunis M. J. (2001). The small ubiquitin-like modifier-1 (SUMO-1) consensus sequence mediates Ubc9 binding and is essential for SUMO-1 modification. J. Biol. Chem. 276, 21664–21669. 10.1074/jbc.M100006200 11259410

[b19] Schulz S. Chachami G. Kozaczkiewicz L. Winter U. Stankovic-Valentin N. Haas P. Hofmann K. Urlaub H. Ovaa H. Wittbrodt J. (2012). Ubiquitin-specific protease-like 1 (USPL1) is a SUMO isopeptidase with essential, non-catalytic functions. EMBO Rep. 13, 930–938. 10.1038/embor.2012.125 22878415 PMC3463963

[b20] Selamat W. Jamari I. Wang Y. Takumi T. Wong F. Fujii R. (2009). TLS interaction with NMDA R1 splice variant in retinal ganglion cell line RGC-5. Neurosci. Lett. 450, 163–166. 10.1016/j.neulet.2008.12.014 19103256

[b21] Seufert W. Futcher B. Jentsch S. (1995). Role of a ubiquitin-conjugating enzyme in degradation of S- and M-phase cyclins. Nature 373, 78–81. 10.1038/373078a0 7800043

[b22] Shin E. J. Shin H. M. Nam E. Kim W. S. Kim J. H. Oh B. H. Yun Y. (2012). DeSUMOylating isopeptidase: a second class of SUMO protease. EMBO Rep. 13, 339–346. 10.1038/embor.2012.3 22370726 PMC3321169

[b23] Tatham M. H. Jaffray E. Vaughan O. A. Desterro J. M. Botting C. H. Naismith J. H. Hay R. T. (2001). Polymeric chains of SUMO-2 and SUMO-3 are conjugated to protein substrates by SAE1/SAE2 and Ubc9. J. Biol. Chem. 276, 35368–35374. 10.1074/jbc.M104214200 11451954

[b24] von Schantz M. Lucas R. J. Foster R. G. (1999). Circadian oscillation of photopigment transcript levels in the mouse retina. Brain Res. Mol. Brain Res. 72, 108–114. 10.1016/S0169-328X(99)00209-0 10521605

[b25] Wei W. Yang P. Pang J. Zhang S. Wang Y. Wang M. H. Dong Z. She J. X. Wang C. Y. (2008). A stress-dependent SUMO4 sumoylation of its substrate proteins. Biochem. Biophys. Res. Commun. 375, 454–459. 10.1016/j.bbrc.2008.08.028 18708028

[b26] Wilkinson K. A. Henley J. M. (2010). Mechanisms, regulation and consequences of protein SUMOylation. Biochem. J. 428, 133–145. 10.1042/BJ20100158 20462400 PMC3310159

[b27] Wilson V. G. Rangasamy D. (2001). Intracellular targeting of proteins by sumoylation. Exp. Cell Res. 271, 57–65. 10.1006/excr.2001.5366 11697882

[b28] Wright A. F. Reddick A. C. Schwartz S. B. Ferguson J. S. Aleman T. S. Kellner U. Jurklies B. Schuster A. Zrenner E. Wissinger B. (2004). Mutation analysis of NR2E3 and NRL genes in Enhanced S Cone Syndrome. Hum. Mutat. 24, 439 10.1002/humu.9285 15459973

