# Supplementary material for: mRNA expression analysis of the SUMO pathway genes in the adult mouse retina
Source: Biol Open. 2015 Jan 23;4(2):224–32. doi: 10.1242/bio.201410645 (PMC4365491; doi:10.1242/bio.201410645)
Supplement: Supplementary Material [file supp_4_2_224__index.html]

mRNA expression analysis of the SUMO pathway genes in the adult mouse retina — Supplementary Material 

# mRNA expression analysis of the SUMO pathway genes in the adult mouse retina

## bio.201410645 Supplementary Material

**Files in this Data Supplement:**

- Supplementary Material - Víctor Abad-Morales et al. doi: 10.1242/bio.201410645
- Table S1 - Sequences of primer pairs used for *in situ* hybridization (left) and qPCR on retina cDNA (right), including all SUMO substrates and enzymes, plus the reference genes *Gapdh*, *Rho* and *Cerkl*.
