# Supplementary material for: mRNA expression analysis of the SUMO pathway genes in the adult mouse retina
Source: Biol Open. 2015 Jan 23;4(2):224–32. doi: 10.1242/bio.201410645 (PMC4365491; doi:10.1242/bio.201410645)
Supplement: Supplementary Material [file supp_bio.201410645_bio.201510645-s1.pdf]

Biology Open (2015) 000, 1–9 doi:10.1242/bio.201410645

Supplementary Material
Víctor Abad-Morales et al. doi: 10.1242/bio.201410645

Table S1: See supplementary webpage

Biology Open
