# Supplementary material for: mRNA expression analysis of the SUMO pathway genes in the adult mouse retina
Source: Biol Open. 2015 Jan 23;4(2):224–32. doi: 10.1242/bio.201410645 (PMC4365491; doi:10.1242/bio.201410645)
Supplement: Supplementary Material [file supp_bio.201410645_bio201410645_Table_S1.docx]

**Table S1.** Sequences of primer pairs used for in situ hybridization (left) and qPCR on retina cDNA (right), including all SUMO substrates and enzymes, plus the reference genes *Gapdh*, *Rho* and *Cerkl*.

| ***In situ* hybridization** | | | **RealTime PCR** | | |
| --- | --- | --- | --- | --- | --- |
| **Gene Symbol** | | **Sequence (5′-3′)** | **Gene Symbol** | | **Sequence (5′-3′)** |
| *Sumo1* | Forward | tccctgcagccgcggtgt | *Sumo1* | Forward | tccctgcagccgcggtgt |
| *Sumo1* | Reverse | ccgtttgttcctgataaacttc | *Sumo1* | Reverse | gggggtctccgcaccact |
| *Sumo2* | Forward | gctccctcagtgcggacc | *Sumo2* | Forward | gctccctcagtgcggacc |
| *Sumo2* | Reverse | tagtagacacctccagtctgc | *Sumo2* | Reverse | tcagtcttgactccttccttg |
| *Sumo3* | Forward | accgttgtcctgacctgtgcta | *Sumo3* | Forward | accgttgtcctgacctgtgcta |
| *Sumo3* | Reverse | cagcacagtccgtgagaatgag | *Sumo3* | Reverse | agaatccttcggtgactctggc |
| *Sae1* | Forward | aagcgtactgcccccgactac | *Sae1* | Forward | gggcaccatgtgtgtctagtgc |
| *Sae1* | Reverse | cacttgcagggcagctgataac | *Sae1* | Reverse | cacttgcagggcagctgataac |
| *Sae2* | Forward | tgaagatgctgccaaaagcatt | *Sae2* | Forward | gagcagatggaagatccagatga |
| *Sae2* | Reverse | ttggttccgaccagaccaag | *Sae2* | Reverse | ttggttccgaccagaccaag |
| *Ubc9* | Forward | cgagcacaagcgaagaagtttg | *Ubc9* | Forward | gcttccatcttggctgtgtgac |
| *Ubc9* | Reverse | cccaggtttctgagaaggacga | *Ubc9* | Reverse | cccaggtttctgagaaggacga |
| *Cbx4* | Forward | taccagggcggccacaag | *Cbx4* | Forward | gcaggcagtgaaaatcaagtcg |
| *Cbx4* | Reverse | ctcctcggcagcacgttt | *Cbx4* | Reverse | ctcctcggcagcacgttt |
|  | | | *Egr2* | Forward | gctccctctgcacctccatct |
|  |  |  | *Egr2* | Reverse | gtcctccaatggcgctgttac |
| *Hdac4* | Forward | tctgctctgctgggaaacgag | *Hdac4* | Forward | tagcccgaagctgctgttctct |
| *Hdac4* | Reverse | gacgcaggagtgatacgggtaa | *Hdac4* | Reverse | gacgcaggagtgatacgggtaa |
| *Hdac7* | Forward | cagaacagatcgcgcttcaaat | *Hdac7* | Forward | tctggtgtccacacccctagc |
| *Hdac7* | Reverse | tagtggttatgggtggcccagt | *Hdac7* | Reverse | tagtggttatgggtggcccagt |
| *Mms21* | Forward | ccgttacatatccttcagtggcata | *Mms21* | Forward | ccgttacatatccttcagtggcata |
| *Mms21* | Reverse | ctgttgcttgaactgcacgaactt | *Mms21* | Reverse | ccagagctgatacaggattggaa |
| *Mul1* | Forward | gtgcatggtgcttgcttcacta | *Mul1* | Forward | cctggccttgctgatgtagttg |
| *Mul1* | Reverse | atcctcttgacccacgccatct | *Mul1* | Reverse | atcctcttgacccacgccatct |
| *Pias1* | Forward | gcctcgcctgtgtcccgca | *Pias1* | Forward | taagtgcaggagggagcacatc |
| *Pias1* | Reverse | tgttggaagacacaaggctgct | *Pias1* | Reverse | tgttggaagacacaaggctgct |
| *Pias2** | Forward | gcaggatgttgcaggagacg | *Pias2* | Forward | cacaaagcagtccaaccaaagg |
| *Pias2** | Reverse | gcagctggacttggactgtgta | *Pias2* | Reverse | tgaaggtggaatagcagcagga |
| *Pias2** | Forward | tgggcatctgaaattggaaaga |  |  | |
| *Pias2** | Reverse | tgaaggtggaatagcagcagga |  |  |  |
| *Pias3* | Forward | gcgcagtccaggagggaat | *Pias3* | Forward | gcgcagtccaggagggaat |
| *Pias3* | Reverse | gagccacaatgctgctgacac | *Pias3* | Reverse | tgatgagctttcgatggtcaag |
| *Pias4* | Forward | ggtgaagatgcgcctctcg | *Pias4* | Forward | ggtgaagatgcgcctctcg |
| *Pias4* | Reverse | acgtcggctcccgtcttg | *Pias4* | Reverse | cattcatttgcaggtagaacacag |
| *RanBP2* | Forward | gcgacttcacattttcatcacca | *RanBP2* | Forward | tcacggtccagggaagtctgta |
| *RanBP2* | Reverse | tcctccatctgtctcgtgactttt | *RanBP2* | Reverse | tcctccatctgtctcgtgactttt |
| *Rasd2* | Forward | ccctgtgcccattctcttgttt | *Rasd2* | Forward | gatgccccagccaggttc |
| *Rasd2* | Reverse | gtggtttctccagggacactga | *Rasd2* | Reverse | gtggtttctccagggacactga |
| *Tls* | Forward | cggtggtggttatggcaatc | *Tls* | Forward | cggtggtggttatggcaatc |
| *Tls* | Reverse | gctgtccagttttcttgtttgtc | *Tls* | Reverse | ggcccccacgatcctgtt |
| *Topors* | Forward | cagacagagagacaaaacacaagagg | *Topors* | Forward | caaactgtggagaattgtgactcg |
| *Topors* | Reverse | ggcaaaggtgctttcttctcttacat | *Topors* | Reverse | ggcaaaggtgctttcttctcttacat |
| *Traf7* | Forward | aggagattgccttcctgcgttc | *Traf7* | Forward | aggagattgccttcctgcgttc |
| *Traf7* | Reverse | agagcgccagcacaataccat | *Traf7* | Reverse | tcaaggctcttctctagctggtca |
| *Desi1* | Forward | ccttcctggactccattcagat | *Desi1* | Forward | ccttcctggactccattcagat |
| *Desi1* | Reverse | cccagactgtgactcttgctttc | *Desi1* | Reverse | ctgttagctttggccattgggt |
| *Desi2* | Forward | cactggcctgaggaaaacgaa | *Desi2* | Forward | gaaagagattcctcgctggatca |
| *Desi2* | Reverse | cttgtgtgctgagctgctgaaa | *Desi2* | Reverse | tggcaggcaactctgtagaaag |
| *Senp1* | Forward | gagggcccccagaagtactga | *Senp1* | Forward | gagggcccccagaagtactga |
| *Senp1* | Reverse | ccatcttcagctgacagcatc | *Senp1* | Reverse | ctaaccgccaaggtccttcttc |
| *Senp2* | Forward | gggctgcttgaaaccctgtt | *Senp2* | Forward | gggctgcttgaaaccctgtt |
| *Senp2* | Reverse | cagttcgcactccagcattag | *Senp2* | Reverse | gcatttgttcttgtatggggtga |
| *Senp3* | Forward | cgagctctccgaccctctcata | *Senp3* | Forward | cgagctctccgaccctctcata |
| *Senp3* | Reverse | tggggtccaccttagtccatct | *Senp3* | Reverse | ggtgctttttgagtagagcagca |
| *Senp5* | Forward | ctggaggtccactggtctctcat | *Senp5* | Forward | ctcgtgtgcggaagaggatcta |
| *Senp5* | Reverse | tctggagtccctgctgagtgag | *Senp5* | Reverse | tctggagtccctgctgagtgag |
| *Senp6* | Forward | ccttgtatcctccttatggactca | *Senp6* | Forward | gtggctcagagtgacgaggag |
| *Senp6* | Reverse | ccccaagagtaaatgctgtgtctg | *Senp6* | Reverse | ccccaagagtaaatgctgtgtctg |
| *Senp7* | Forward | ttcctcgccatgtaataaagacc | *Senp7* | Forward | actgggtgttgatgggaggtc |
| *Senp7* | Reverse | gatgtgacttgtcaacacacctgac | *Senp7* | Reverse | gatgtgacttgtcaacacacctgac |
| *Senp8* | Forward | cccttccttgttggcttattcc | *Senp8* | Forward | tggtgtgcaggaagacttgaac |
| *Senp8* | Reverse | ccactcactcagggaaaccaga | *Senp8* | Reverse | ccactcactcagggaaaccaga |
| *Uspl1* | Forward | aacgagaaaccacagcgtcttc | *Uspl1* | Forward | aacgagaaaccacagcgtcttc |
| *Uspl1* | Reverse | gccaggagatctgtgcctga | *Uspl1* | Reverse | ctgatcttctttccgaggctga |
| *Rho* | Forward | gcccttctccaacgtcacag | *Rho* | Forward | gcccttctccaacgtcacag |
| *Rho* | Reverse | gcagcttcttgtgctgtacgg | *Rho* | Reverse | gcagcttcttgtgctgtacgg |
|  | | | *Gapdh* | Forward | tgacaatgaatacggctacagcaa |
|  |  |  | *Gapdh* | Reverse | tactccttggaggccatgtagg |
|  |  |  | *Cerkl* | Forward | tcaattttccctttgtggagac |
|  |  |  | *Cerkl* | Reverse | catgcctggtgccacctggt |
|  |  |  | *Crx* | Forward | ccttccagcggaatcactcttt |
|  |  |  | *Crx* | Reverse | tgctgttgcttgctgagagcta |
|  |  |  | *Nr2e3* | Forward | gccttggccagtgcagagac |
|  |  |  | *Nr2e3* | Reverse | gccttcaggcaggcaaactc |
|  |  |  | *Nrl* | Forward | gggtccgacgaccacacac |
|  |  |  | *Nrl* | Reverse | atctacccagccacctccaaac |

* The asterisk indicates that two riboprobes (generated from two primer pairs covering different gene regions) were simultaneously used in the same in situ hybridization assay.
